# Community Ecosystem Mapping: A Foundational Step for Effective Community Engagement in Research and Knowledge Mobilization

**DOI:** 10.1177/21501319231205170

**Published:** 2023-10-16

**Authors:** Tanvir C. Turin, Mashrur Kazi, Nahid Rumana, Mohammad A. A. Lasker, Nashit Chowdhury

**Affiliations:** 1 Department of Family Medicine, Cumming School of Medicine, University of Calgary, Calgary, AB, Canada; 2 Department of Community Health Sciences, Cumming School of Medicine, University of Calgary, Calgary, AB, Canada; 3 Community Scholar and Citizen Researcher, Calgary, AB, Canada; 4 Community Champion and Citizen researcher, Calgary, AB, Canada

**Keywords:** community, patient-centeredness, community health, health promotion, prevention

## Abstract

Community engagement is a key strategy for achieving various goals, such as social and environmental change, sustainable development, health promotion, and community building. It involves collaborations and partnerships with the community that help mobilize resources, impact systems, rectify partner dynamics, and function as catalysts for modifying policies, programs, and practices. It also ensures mutual trust among all parties involved, giving community members greater personal agency and involvement potential. We have learned a range of practical aspects of community engagement with communities, particularly with immigrant/racialized communities, by running a community-engaged program of research on the health and wellness issues of immigrant/racialized communities in Calgary, Canada. In this article, we focus on a crucial early step of community engagement—understanding the community ecosystem. The community ecosystem refers to its human, social, and cultural makeups. Understanding this ecosystem requires conscious efforts to comprehend the demography, participate in socio-cultural events, identify community spots, reach out to hard-to-access groups, find the community champions and communication channels/organizations, and reaching out to them to establish relationships. Understanding the community ecosystem allows us to identify the pivotal factors, key actors, and pulse of the community that we are engaging with. This enables us to build mutual trust and goals for research and knowledge mobilization. Subsequently, an empowered, continual, and collaborative partnership becomes possible, resulting in sustained and desirable outcomes.

## Introduction

Community engagement is identified as a crucial approach for social and environmental changes, sustainable development, health promotion, community building, and much more.^1-4^ The community engagement process has been described as “*a powerful vehicle for bringing about environmental and behavioral changes that will improve the health of the community and its members. [It] often involves partnerships and coalitions that help mobilize resources and influence systems, change relationships among partners, and serve as catalysts for changing policies, programs, and practices*.”^5^ Due to the multifaceted nature of community engagement, the process has proven to be beneficial in a variety of contexts including philanthropy, community-based research, social advocacy, community organizing, policy making, and more.^1-5^ Community engagement allows the building of mutual trust between all stakeholders involved, therefore empowering members of the community with personal agency and capacity for engagement.^1,2,4^ Implementing community engagement for research is beneficial for both members of the community and researchers.^3,4^ Taking this approach allows for creating an empowered, continual, and collaborative partnership between 2 parties, thereby leading to sustained and desirable outcomes. Involving the community in the full gamut of the research process is the core principle of Community-Based Participatory Research (CBPR) or Community-Engaged Research (CEnR).^6,7^ By doing so, researchers can shift the dynamic of the traditional researcher-participant relationship (i.e., researchers driving the research process with minimal assistance from the community) to a more symmetric partnership, where the community and researchers have equal decision-making power regarding the research process.^3,6,7^

**Summary Box**

1. An exhaustive endeavor to learn about an immigrant/racialized community is crucial for the development of effective community engagement strategies for equitable and empowered involvement of the community in research.
2. Understanding the community ecosystem is a prerequisite to developing engagement strategies. A community ecosystem includes its human, social, and cultural makeups of a community.
3. This entails making deliberate efforts to understand the community demographics, taking part in sociocultural activities, locating community spots, identifying difficult-to-access groups, and recognizing and reaching out to the community champions, communication channels, and organizations.
4. Initially, this may seem a rather huge task, however, once a better understanding of the community ecosystem is achieved it leads to sustainable strategies of community engagement followed by an effective and meaningful program of research.
5. The researchers need to be mindful that the community ecosystem is an ongoing process. Especially, due to the immigration patterns in Canada the demographics and dynamics of the communities are always evolving, thereby requiring regular updating of the knowledge of the community ecosystem.

Researchers need to strive to diligently engage the community in meaningful ways rather than merely partake in tokenism. The National Academy of Medicine in the United States recommends that researchers put emphasis on strengthening partnerships and alliances with community members to achieve effective and meaningful community engagement.^8^ This is particularly critical for conducting research involving under-resourced and vulnerable communities such as immigrant/racialized communities. Research is often not the priority of the community members, especially those who are predisposed to additional life struggles such as immigrant communities with resettlement challenges, racialized communities with racial discrimination, and the various societal challenges of the 2SLGBTQI+ [Two-Spirit, lesbian, gay, bisexual, transgender, queer, intersex, or who use other terms related to gender or sexual diversity] communities. To some extent, it is the researchers’ responsibility to identify the community needs and engage them to develop a sense of belonging regarding the research process and to build trusting relationships. Despite wide recognition of the importance of community engagement with immigrant/racialized communities, little guidance is available about what steps can be taken during a community engagement process toward building a sustainable relationship with these communities.

## Community-Engaged Research With an Immigrant/Racialized Community

We developed a community-engaged program of research on the health and wellness issues of immigrant/racialized communities in Calgary. As a part of this, we worked with a South-Asian community, the Bangladeshi-Canadian community, on primary care access issues^9-12^ since 2013. Taking a community engaged approach, our work started with reaching out to the community members in an effort to build a relationship with the community so that we could conduct research together.^13-15^ We ensured that the engagement efforts were embedded within a participatory approach and provided all-out efforts to build a sustainable relationship with community members. Regarding community engagement, we realized from our initial experiences we need to know the community very well to devise the engagement strategies to conduct a thorough community engagement process. In this article, we reflect on this aspect of an applied community engagement strategy.

## Understanding the Community Ecosystem

**Figure 1.**
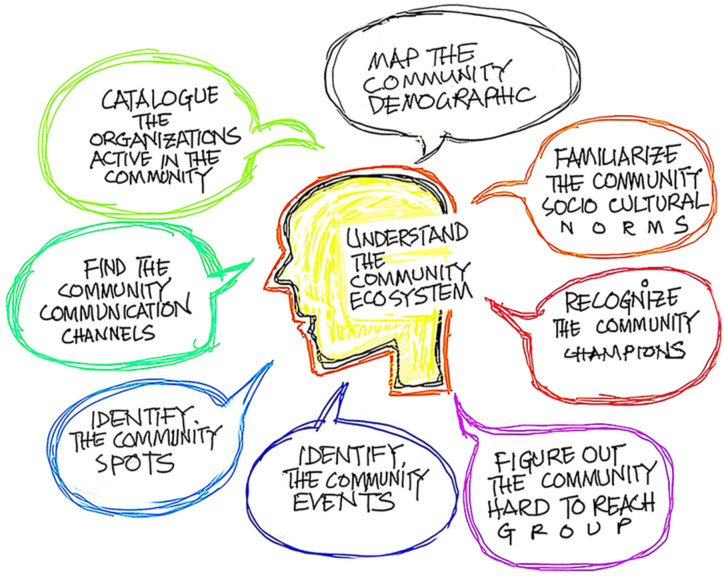
Elements of understanding the community ecosystem.

The first thing that needs to be done for strategic community engagement is to understand the community’s ecosystem. An immigrant/racialized community’s ecosystem includes its human, social, and cultural makeup. For instance, the demographic distribution and interactions among various community members with different capacities and roles are important. These include community leaders, general members, the community’s internal support systems and mechanisms, and relationships among different segments of the community, such as those between different faiths and between affluent and economically disadvantaged individuals. Other interactions involve neighborhood characteristics (eg, crime rates and environment), the interactions with other communities, and the relationship and attitude(s) with the host country and native people that collectively define the community.^16^ Without having a properly informed idea about the community we plan to engage, the engagement efforts face the risk of falling short, thereby becoming confined to a smaller section of the extended community. In our experience, the efforts for this need to happen across a number of elements (Figure 1).

### Map the Community Demographic

Researchers need to understand the community demographics before attempting an engagement effort within the community. The distribution of age, gender, socio-economic status, education or professional background, current job domain, recency of immigration, routes of immigration, and religious affiliation-based distribution are some aspects to consider. These ultimately help to gain an understanding of the community structure. We found that information about these issues from official sources is very limited, especially about the racialized/immigrant communities. In the context of our work, we endeavored to collect information from a range of sources, which helped to shape our understanding. We looked for whatever information was available through various federal, provincial, or local government data sources such as Census Canada data,^17^ Calgary Civic Census data,^18^ and more. We extended our search to the published literature and reports. We also conferred with the community-connected elders (in terms of length of stay in Calgary), leadership position holders in Bangladeshi-Canadian socio-cultural organizations, as well as community members who work in different community-serving organizations such as immigrant service provider organizations (ISPO) or faith-based organizations. Our experience of lack of availability of data is similar to a recent synthesis report that highlighted the lack of data for research on visible minorities.^19^

### Familiarize the Community Socio-Cultural Norms

Social-cultural norms or customs are expectations of behavior and thoughts based on shared beliefs within a specific cultural or social group. Cultural humility and cultural competence are 2 important parts of any community engagement strategy, through which we demonstrated our cultural humility to the community. With their help, we gradually gained a certain level of competence, particularly being able to have community scholars and citizen researchers from the community on our research team. Familiarity with the socio-cultural norms of the community conferred advantages that allowed research design that recognizes cultural values. To illustrate, in the Bangladeshi-Canadian community, it is important to know about sociocultural norms like not requesting a meeting during prayer time or accommodating prayer breaks during the meetings, interaction style (e.g., shaking hands/hugging when meeting men and not doing so while interacting with women), communication/linguistic preference (greeting/goodbye words, or different ways of addressing people from different age groups or gender), etc. By having researchers belonging to the Bangladeshi-Canadian community on our research team, we gained insider perspectives^20^ on the socio-cultural norms that ultimately furthered our community engagement strategy remarkably. When we extended our research toward another immigrant/racialized communities, we emphasized efforts to understand the socio-cultural norms of those communities as well.

### Recognize the Community Champions

Gaining support and developing relationships with the community champions (activists, influencers, and leaders) is critical for the success of a community-engaged program of research. This is because these relationships go a long way to enhance the process of community engagement. Based on our experience, we defined community activists as individuals from the community who are proactive in enacting social change and who demonstrate natural leadership qualities. We defined community influencers as those who are well-known and revered in their community as celebrities or beloved figures, such as musicians, artists, and language instructors. Lastly, we defined a community leader as someone who has an official leadership role in any kind of community organizations.

We reached out to the community champions and asked them to allow us some time to meet and showcase our ideas regarding community engagement and research. We met at coffee shops or community centers and asked for their opinion and direction on how best to go ahead with our plan. We had an interesting observation at the initial stage: We saw that most of the community spearheads are men due to the socio-cultural norm in the community. As a result, we devised an unorthodox plan to identify and connect with outwardly less visible women champions of the community. We began by approaching a few women we personally knew and informing them about our efforts. Then, by taking a snowball approach, we requested them to put us in touch with their women colleagues, friends, or family members they thought we should be contacting. We found that the women community spearheads were more enthusiastic about taking part in the community engagement and research process and were also proactive about conveying the information of the project to other potential community members. As women often play the role of “social glue” in the social relationships within the community, this approach of engaging women boosted the outreach of the project. Over time, some women evolved into being more engaged than others and became core members of the research team (now identified as community scholars and citizen researchers).

### Figure out the Community’s Hard-to-Access Groups

Guided by the social justice perspective and principles of equity, diversity, and inclusion, we wanted to understand the different hard-to-access subgroups of the community (such as 2SLGBTQI+ people, people with disabilities, and religious minorities), who may or may not be visible in the initial demographic examination. This is important because by knowing about them we can make active efforts to reach those inaccessible community subgroups through our community-engaged program of research. This has been the most difficult part of our engagement and unfortunately, we cannot claim much success in this regard. We believe that the generic community engagement strategy is not effective in reaching the hard-to-reach populations. Instead, we may need to devise focused and tailored strategies for the specific sub-groups. Nevertheless, due to our overall community engagement activities, we think they are aware of our intentions and efforts in the general community engagement. And hopefully it increases the likelihood of engaging the hard-to-reach groups as we continue our efforts.

### Identify the Community Events

A good strategy is also to have knowledge about various events that take place in the community regularly. For example, in the Bangladeshi-Canadian community in Calgary—there are 2 major festivals that happen in a year. One is celebrating the Bangla New Year’s Day, which occurs in the Spring. Another occurs during the Fall and it revolves around cultural activities related to the harvesting seasons in Bangladeshi tradition. Community members flock in huge numbers to take part in the festivities. Also, there are religious festivities of 2 Eid celebrations and multiple Puja celebrations, both of which are celebrated on relatively larger scales. Knowing about these occasions allowed us to be present at events where we could interact with the community members or show them our presence. It’s practically impossible to show up to all of the festivals, rather we chose a couple of these events where we could maximize our engagement efforts based on our resources, time, and available manpower.

### Inventory the Community Spots

Finding the spots that the community visited as part of their everyday lives was another crucial factor that assisted us in our engagement activities, especially for the research portion. We took an inventory of ethnic Bangladeshi community member-owned grocery stores, restaurants, businesses (travel agent offices, immigrant consultant offices, and boutique shops), and places of worship such as mosques and temples. This helped us plan outreach and knowledge dissemination strategies toward the community members. For instance, with the owners’ consent, we frequently posted research flyers, brochures, and event information in various locations.

### Find the Community Communication Channels

In our experience, we found that the racialized/immigrant communities tend to lie outside of the traditional mainstream research communication channels. These communities are not a significant portion of the audience of mainstream print or TV media. Rather, at the societal level, they are more used to “ethnic media” or “social networking sites.” Social networking sites have already become a communication channel of preference during this era of digitalization. During the COVID-19 pandemic era innovative ways of communication through social media exploded. Indeed, social media has increasingly become an alternative to even ethnic print or TV media for the racialized/immigrant community to acquire information. Also, social connectedness through social networking sites have received momentum. For our engagement purposes, we identify the ethnic media, and social networking site channels which could be used for the engagement purposes. For example, the Bangladeshi-Canadian community uses Facebook as their predominant mode of social connectedness and communication, and our outreach strategy has a special focus on this communication channel choice of the community.

### Catalogue the Organizations Active in the Community

Many organizations are active in immigrant/racialized communities and engage in a variety of activities, including cultural, educational, religious, developmental, and charitable enterprises. These organizations also have a variety of organizational structures, including for-profit entities, entities registered as charities, or not-for-profit organizations, while others have an informal structure. Also, members of immigrant communities tend to maintain social networks through their previous or current professional or educational networks. There are a number of sociocultural groups within the Bangladeshi-Canadian community in Calgary. For instance, professional associations (eg, agriculturalist association), alumni groups (eg, University of Dhaka alumni), religious affiliation (eg, Islamic study group and Hindu prayer group), and cultural activities (eg, Bangla cultural group and theater group). The goal of contributing to the general social development of society is what unites these groups. We made contact with these groups in an effort to establish relationships and earn their conviction and support before beginning research on issues and projects at the grassroots community level.

## Contemplate That All of the Aspects of a Community Ecosystem Are Dynamic in Nature

It is important to note that all the aforementioned factors need to be part of an ongoing process. The community itself is dynamic, and various factors are constantly evolving over time. Due to shifting immigration patterns in Canada, the demographics of the communities continue to change over time. New people always step into positions when the community’s leadership changes, and the champions go on to new endeavors. While the old connections disappear through attrition due to normal change, it’s critical that new connections be made by the researchers with similar effort and intention. Therefore, it is crucial to continuously be aware of the dynamics of the community in order to plan for effective and meaningful community participation.

## Strengths and Challenges of the Approach

Community ecosystem mapping involves gathering, analyzing, and visually representing data about the sociocultural fabric of a community prior to commencing research endeavors. This approach, while advantageous in several aspects, also presents certain challenges in the realm of community-based research.

This method offers researchers valuable insights to tailor their studies to address the specific needs, strengths, opportunities, and obstacles within the community. By comprehending the issues and potential solutions that hold significance to the community, researchers can harmonize their research objectives and methodologies accordingly. Moreover, it facilitates the establishment of trust, rapport, and collaborative relationships with community stakeholders. Through their involvement in data collection and analysis, their wisdom and perspectives are respected, fostering a foundation of cooperation. Furthermore, community ecosystem mapping enables researchers to pinpoint pivotal junctures for systemic change and societal influence. This entails identifying the interdependencies, interplays, and dynamics within the community structure, providing a canvas to explore the ways in which research outcomes can influence, or be shaped by, these dynamics.

On the other hand, this approach does present some challenges. It requires substantial resource and time investments which is often overlooked while conducting a research project. Its applicability can also be constrained in multifaceted and intricate communities, as it might not capture the entirety of subtleties, perspectives, and conflicts present within and among different factions—such as ethnicity, gender, age, socio-economic status, religion, and the like. Moreover, community ecosystem mapping is susceptible to the biases, reach, and assumptions of the persons who are undertaking it. Their distinct agendas, expectations, and interpretations could influence the data collected and analyzed. Hence, researchers employing this approach need to remain cognizant of its merits and limitations, effectively planning their research endeavors in accordance with its nuances.

## Conclusion

While working for our program of research we realized that our initial understanding of the community ecosystem was more incomplete than we had originally considered. Being a member of a community, organization, or group does not automatically mean that the researchers know or have access to all aspects of the community, organization, or group for research purposes. Moreover, we also recognized that no singular source is good enough to get community ecosystem-related information for ethnic minority communities. We need to strategize and work on that extensively. An important factor to note is that the mapping of the community ecosystem is an iterative process and needs to be approached as if we are creating a living document. Certainly, time is needed for initial exploration, and this is definitely time consuming, but the yield of the process is very helpful for any community engaged program of research. Furthermore, this exploration creates an avenue for engaging with a diverse spectrum of community members. Keeping the community informed of ongoing findings is consistently valued, contributing to a sense of inclusivity and transparency throughout the process. In the context of understanding a particular community, these aforementioned undertakings can serve as powerful engagement mechanisms, especially when executed through ongoing interactions with community members as the mapping journey unfolds.

## Recommendations

Based on this experience, we recommend that future researchers or practitioners who use this approach need to:

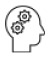
 Plan ahead and allocate sufficient time and resources for the data collection, analysis, and visualization of the community ecosystem.
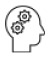
 Remain open to adaptation and flexibility in response to the ever-evolving dynamics within the community. Treat the community ecosystem as a living document, fostering a strategy for regular updates to the mapping, reflecting its current state accurately.
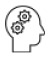
 Solicit feedback and insights from community stakeholders throughout the entire process, valuing and acknowledging their reservoir of knowledge and perspectives.
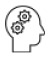
 Acknowledge personal limitations in terms of positionality, social networks, and reach within the community. Maintain awareness of these constraints while engaging in the process.
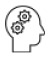
 Articulate the results and implications of the mapping to the community in a manner that is both accessible and resonant, fostering a meaningful understanding.
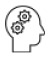
 Leverage the mapping as a foundation for discerning the community’s requirements, strengths, opportunities, and challenges. Align research objectives and methodologies harmoniously with these insights.
